# Supplementary figures and images for: SARS-CoV-2 Infection Prompts IL-1β-Mediated Inflammation and Reduces IFN-λ Expression in Human Lung Tissue
Source: Pathogens. 2022 Nov 21;11(11):1390. doi: 10.3390/pathogens11111390 (PMC9698775; doi:10.3390/pathogens11111390)

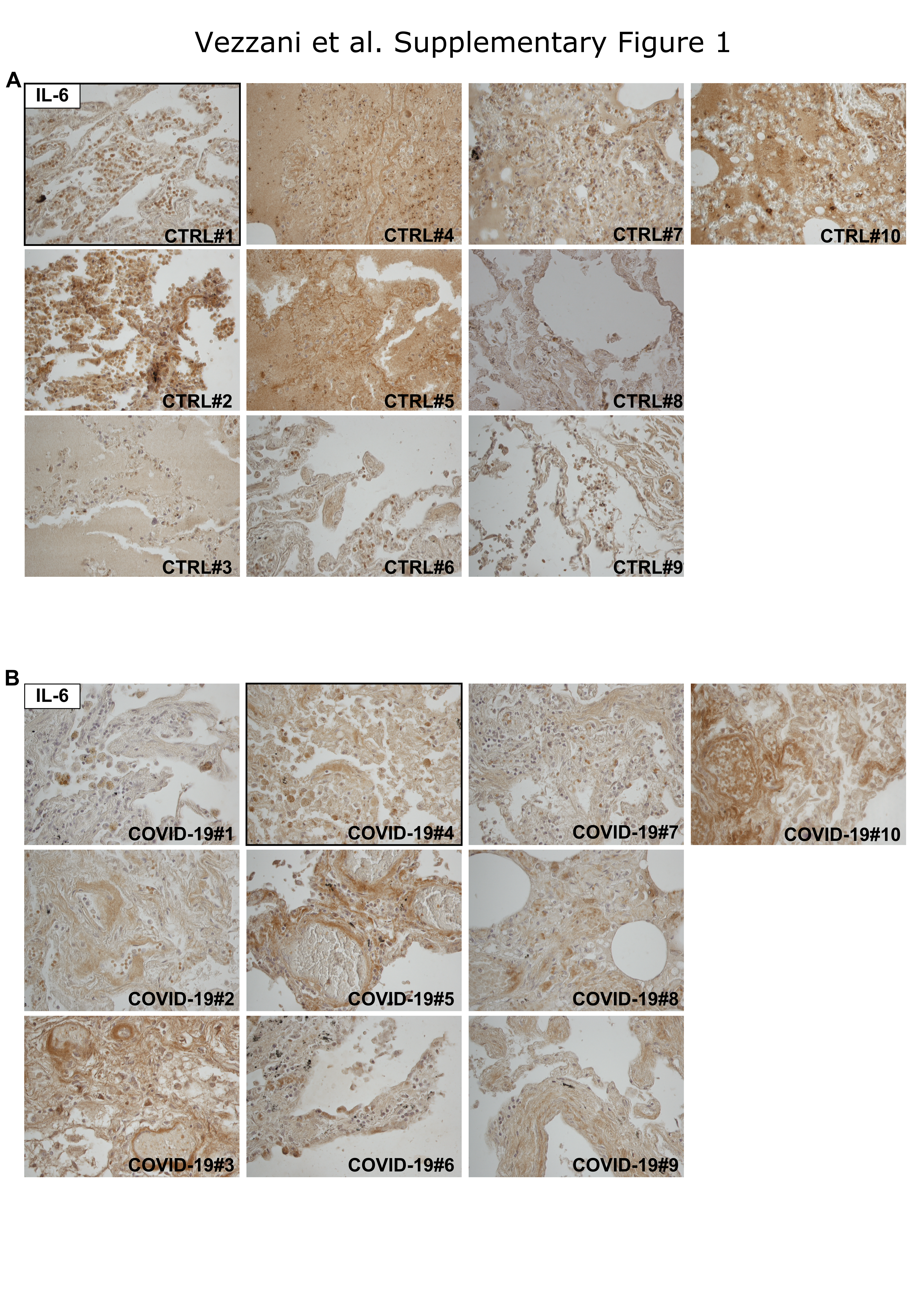

Supplement: Supplementary file 1 [file pathogens-11-01390-s001.zip › Supplementary figures/FIG S1.png]

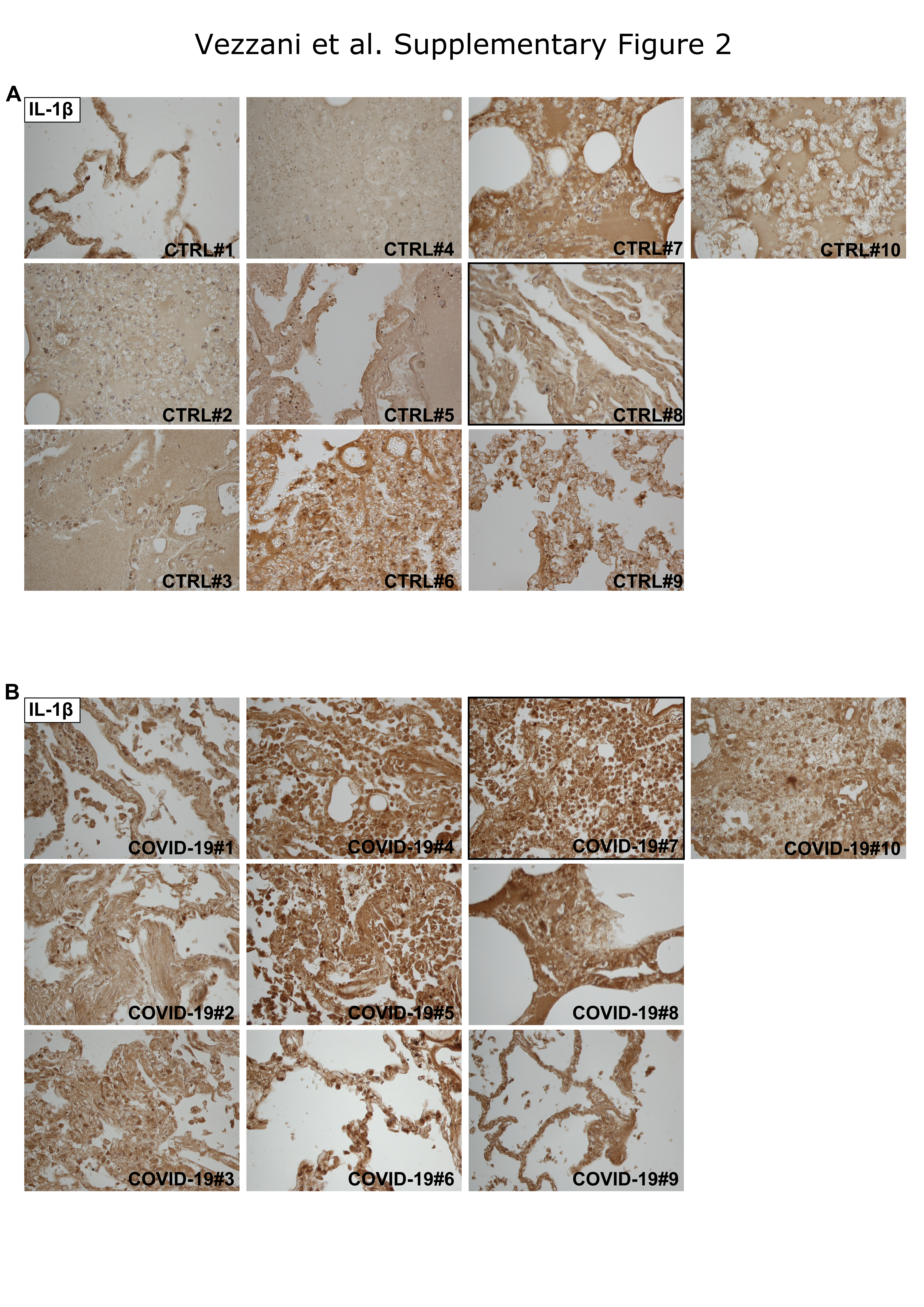

Supplement: Supplementary file 1 [file pathogens-11-01390-s001.zip › Supplementary figures/FIG S2.png]

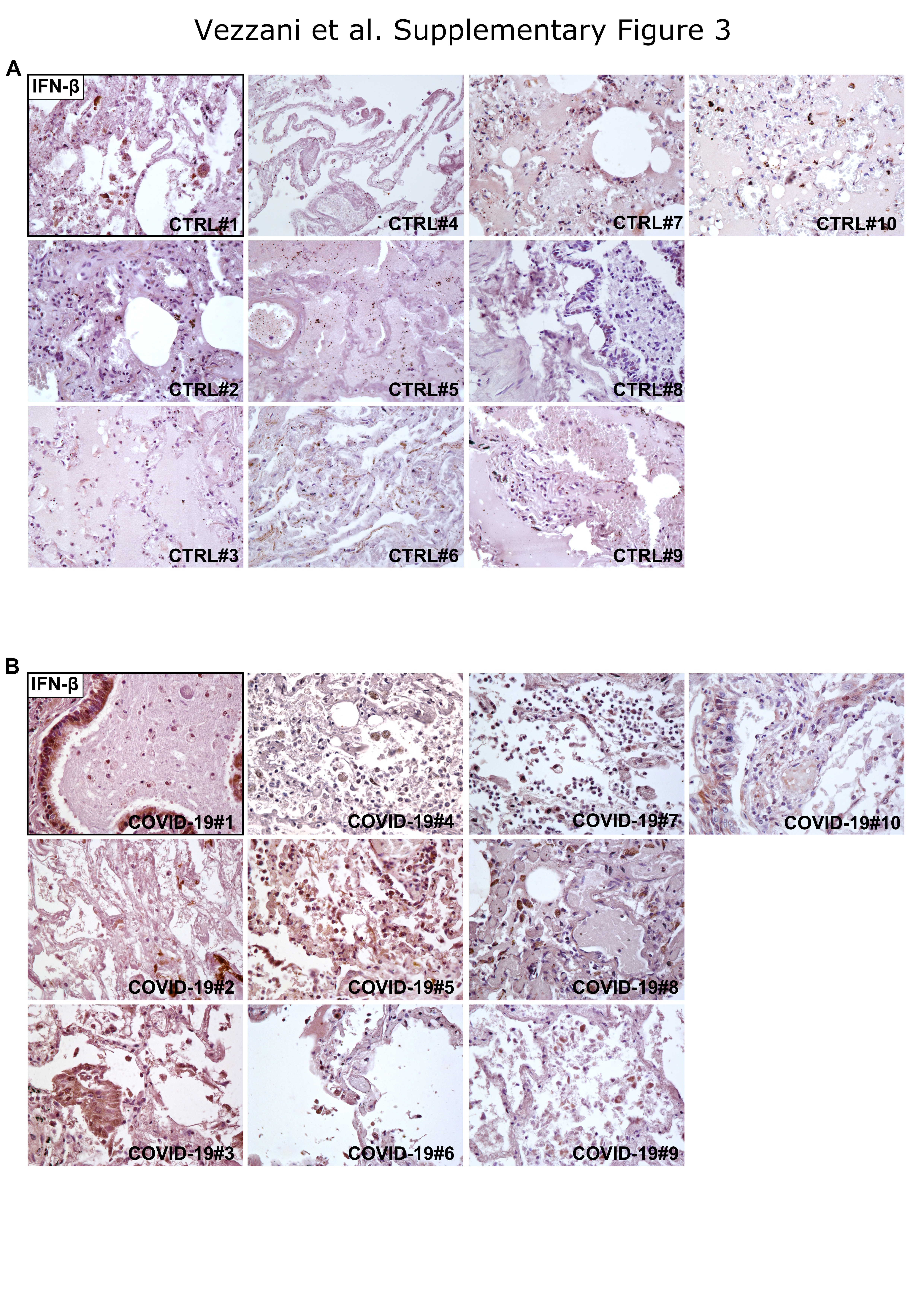

Supplement: Supplementary file 1 [file pathogens-11-01390-s001.zip › Supplementary figures/FIG S3.png]

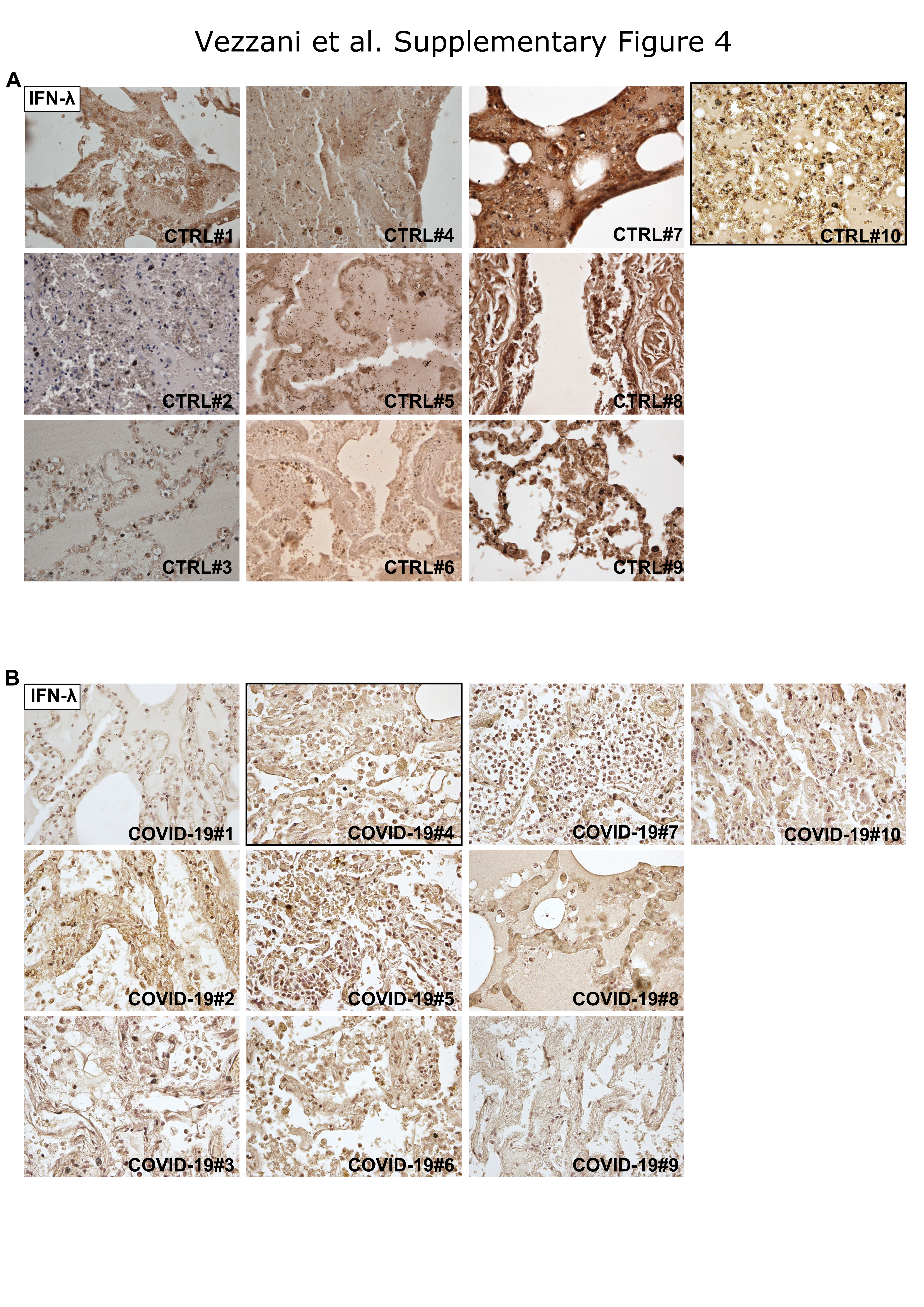

Supplement: Supplementary file 1 [file pathogens-11-01390-s001.zip › Supplementary figures/FIG S4.png]
